# Drug Repurposing of Pantoprazole and Vitamin C Targeting Tumor Microenvironment Conditions Improves Anticancer Effect in Metastatic Castration-Resistant Prostate Cancer

**DOI:** 10.3389/fonc.2021.660320

**Published:** 2021-07-07

**Authors:** Zhoulei Li, Peng He, Yali Long, Gang Yuan, Wanqing Shen, Zhifeng Chen, Bing Zhang, Yue Wang, Dianchao Yue, Christof Seidl, Xiangsong Zhang

**Affiliations:** ^1^ Department of Nuclear Medicine, The First Affiliated Hospital of Sun Yat-Sen University, Guangzhou, China; ^2^ Department of Geriatrics, The First Affiliated Hospital of Sun Yat-Sen University, Guangzhou, China; ^3^ Department of Nuclear Medicine, Klinikumrechts der Isar, Technical University Munich, Munich, Germany

**Keywords:** drug repurposing, proton pump inhibitor pantoprazole, pH value, metastatic castration-resistant prostate cancer, 18F-FDG PET/CT

## Abstract

The effective and economical therapeutic strategy for metastatic castration-resistant prostate cancer (mCRPC) is still requested from patients, who are not available for Lu-177 or Ra-223 treatment. Drug repurposing as a cost-effective and time-saving alternative to traditional drug development has been increasingly discussed. Proton pump inhibitors (PPIs) such as pantroprazole, which are commonly used as antacids, have also been shown to be effective in cancer chemoprevention *via* induction of apoptosis in multiple cancer cell lines. Vitamin C is an essential micronutrient for human body, has been proposed as a potential anti-cancer agent. In this context, have we investigated the combination of vitamin C and pantoprazole for the management of metastatic castration-resistant prostate cancer (mCRPC). Six chosen human adenocarcinoma cell lines were used to investigate the influence of pantoprazole on the microenvironment of cancer cells (extracellular pH and production of exosomes). Tumor growth and tumor 18F-FDG uptake in PC3 xenografts were analyzed following varied treatment. Our *in vitro* Results have suggested that pantoprazole enhanced the cytotoxic activity of vitamin C by regulating pH values and production of exosomes in cancer cells. Moreover, the synergistic effect of pantoprazole and vitamin C was pH-dependent since pantoprazole was more effective at a slightly acidic pH. In vivo, the combined treatment using pantoprazole and vitamin C produced better therapeutic outcomes than treatment with vitamin C or pantoprazole alone, as demonstrated *via* tumor growth and uptake of 18F-FDG. Therefore, we suggest that pantoprazole combined with vitamin C could be as a possible strategy to manage mCRPC.

## Introduction

Prostate cancer is the second most common cancer in men worldwide and the sixth most common cancer in men in China (1, 2). In early stage, when the disease is limited to the prostate, surgical and/or medical castration is the most preferred therapy, but more than 90% of patients develop castration-resistant prostate cancer (CRPC) (1). Although, radiotherapy is an alternative therapeutic choice for patients with CRPC (3), the relapse presented following radiation therapy in 30–50% patients still (4, 5).One new and very effective strategy involves the administration of radio-ligands that target prostate-specific membrane antigen (PSMA), which is overexpressed in prostate cancer cells. For this purpose, both the beta-emitter Lu-177 and the alpha-emitter Ac-225 are coupled to PSMA-617 or PSMA-I&T targeting metastatic prostate cancer cells (6, 7).However, these therapies are not promoted by the China Food and Drug Administration (CFDA). Therefore, the development of an effective, alternative therapeutic strategy for CRPC is still requested. Drug repurposing allows a quicker, cheaper, and probably more efficient translation from the laboratory to the clinic than the development of new drugs (8, 9). In a previous study, we could demonstrate that sulfasalazine, which is used for the treatment of inflammatory arthritis and inflammatory bowel diseases, improves the anticancer effect of pharmacological vitamin C in mCRPC cells (10).

Proton pump inhibitors (PPIs) such as pantoprozole, esomeprazole and omeprazole, which are commonly used as antacids, have also been shown to be effective in cancer chemoprevention *via* induction of apoptosis in multiple cancer cell lines (11, 12). Because of their wide availability and low cost, PPIs are promising candidates for drug repurposing (13). PPIs exert anticancer effects by targeting the tumor microenvironment, is specifically characterized by acidification and hypoxia (14). An acidic extracellular environment induces tissue damage and stimulates the destruction of enzymes in the extracellular matrix (ECM), thus potentiating metastasis and multidrug resistance (MDR) cell phenotypes (15–17). Therefore, targeting the pH value of the tumor microenvironment is considered as an effective strategy for the treatment of cancer. PPIs are commonly used to treat acid-related diseases through disruption of pH homeostasis in tumor cells by targeting V-ATPase (11, 16).

Intravenous administration of a pharmacological dose of vitamin C has been shown to promote the death of therapy-resistant cancer cells in various cancers (18). Reactive oxygen species (ROS), which are constantly formed metabolic products in mammals, can induce concentration-dependent apoptotic cell death (19, 20). Vitamin C has been reported to induce apoptosis of cancer cells through the generation of ROS, including superoxide (O_2_^-^) and H_2_O_2_ (21–23). Furthermore, results of our study suggest that the pH-value of the extracellular environment could be an important contributor to the anticancer effect of vitamin C (24). PPIs have been reported to enhance anticancer effects on melanoma cells through the regulation of extracellular pH, induction of apoptosis and the accumulation of ROS (25, 26). In the current study, we highlight the regulatory effects of anticancer treatment with a combination of vitamin C and pantoprazole on the pH value, the production of exosomes in the tumor microenvironment, and ROS production. In addition, the results of the present study suggest that vitamin C in combination with pantoprazole could be repurposed for patients suffering from mCRPC.

## Methods and Materials

### Cell Lines

The human adenocarcinoma cell lines PC3, DU145, MCF7, SKBR3, OVCAR3 and SKOV3 were purchased from the Cell Bank of the Chinese Academy of Sciences. The cells were cultured in RPMI 1640 medium (PC3, OVCAR3 and SKOV3) (Gibco, Grand Island, NY, USA) or MEM (DU145, MCF7 and SKBR3) (Gibco) containing 10% FBS (Gibco) and 1% penicillin/streptomycin (MRC, Changzhou, China). All cell lines were cultured at 5% CO2 and 37°C.

### Drugs

Vitamin C (Sigma-Aldrich, St. Louis, MS, USA) was solubilized in phosphate buffered solution (PBS, MRC, Changzhou, China) to prepare a 40 mM stock solution and stored at 4°C. For the *in vitro* study, vitamin C was diluted to concentrations of 1, 2, 4, 8 and 16 mM. Chelators that inhibit redox cycling of iron (i.e., desferrioxamine (DFO, Sigma-Aldrich) and diethylenetriamine-pentaacetic acid, (DTPA, Sigma-Aldrich) were solubilized in PBS to prepare 2 mM and 10 mM stock solutions, respectively, and stored at 25°C. Vitamin C was diluted to concentrations between 0 and 8 mM in cell culture medium at pH 6.5 and 7.5 to detect the influence of the cell culture pH value on the therapeutic effect of drugs. The cell culture medium was titrated to different pH values with hydrochloric acid and sodium hydroxide. DFO and DTPA were diluted to 200 μM and 1 mM, respectively, with medium. Pantoprazole (MCE, Monmouth Junction, NJ, USA) was solubilized in distilled sterile water to prepare a 10 mM stock solution and stored at -20°C.

### WST-8 Assay

The WST-8 assay was carried out according to the manufacturer’s instructions (DOJINDO Laboratories, Kumamoto, Japan) to detect the effects of different treatments on cell viability. Briefly, 1×10^4^ cells per well of a 96-well plate were incubated in 100 μL at 37°C, pre-treated with or without pantoprazole (100 μM) for 24 h and then treated with vitamin C (4 mM) for 4 h. Then, 100 µL of WST-8 reagent was added per well, and the cells were incubated for 1-2 h. Absorbance was measured at 450 nm using a Multiskan FC instrument (Thermo Fisher Scientific, Waltham, MA, USA). Cell viability was calculated according to: absorbance of the sample/absorbance of the control (24).

### Detection of Apoptotic Cells *via* Flow Cytometry

Adenocarcinoma cells (5×10^5^/well in 6-well plates in 3 mL) were first incubated with or without pantoprazole (100 μM) for 24 h at 37°C. Then, vitamin C was administered at different concentration. After 16 h, the cells were detached using 0.05% trypsin solution, washed two times with PBS and centrifuged at 1,500 rpm for 5 min. Then, the cells were stained with fluorescein isothiocyanate (FITC)-labelled annexin V (BD Pharmingen, San Diego, CA, USA), counterstained with propidium iodide (PI; BD Pharmingen), resuspended in binding solution (BD Pharmingen), according to the manufacturer’s instructions (BD Pharmingen), and finally analyzed by flow cytometry (CytoFLEX S, Beckman Coulter, Pasadena, CA, USA).

### Detection of Intracellular Reactive Oxygen Species (ROS)

ROS assays were carried out according to the manufacturer’s instructions (Sigma-Aldrich). Briefly, 5×10^3^ cells per well (96-well plate) were incubated at 37°C with or without pantoprazole (100 μM) for 24 h. Then, vitamin C (4 mM) was added for the cell culture for another 4 h. Finally, 100 μL of ROS detection reagent solution diluted in assay buffer was added to each well, and the cells were incubated for 1 h. Florescence intensity (λex=640/λem=675 nm) was measured using a SPECTRAmax M5 instrument (Molecular Devices, San Jose, CA, USA). The relative ROS signal was determined by calculating the ROS level in the cells with regard to cell survival rate determined from the WST-8 assay and standardizing the value to the ROS signal of untreated controls.

### Determination of Cellular pH Change *via* Flow Cytometry

Adenocarcinoma cancer cells (5×10^5^/well in 6-well plates in 3 mL) were seeded and pre-cultured in conditioned medium with adjusted acidity-alkalinity (pH 6.5 or 7.5) with or without pantoprazole (100 μM) for 24 h. After 24 hours of pantroprazole treatment, cells were collected and washed twice with PBS. Then, cells were incubated for 5 minutes at 37°C with 500 μL of pre-warmed PBS containing 1 μM LysoSensor probe. The intracellular pH was detected using flow cytometry (11).

### Determination of the Change in the pH of the Cell Culture Medium With a pH Metre

Cancer cells (4×10^5^ cells/well in 24-well plates) were seeded and pre-cultured in conditioned medium with adjusted acidity-alkalinity (pH 6.5 or 7.5) with or without pantoprazole (100 μM) for 4 h. The pH of the medium was determined in triplicate using a SevenCompact™ev220 pH metre (METTLER TOLEDO, Columbus, OH, USA).

### Purification of Exosomes From Cell Culture Supernatants

PC3 and DU145 cells were cultured with cell culture medium with a pH between 6.5 and 7.5 for two weeks, and then 1.5 – 2.0×10^6^ cells were incubated in 75 cm^2^ flasks until they reached approximately 60–70% confluence. Subsequently, the cells were further incubated with exosome-free medium (Gibco) for 24 h. Then cell culture media was collected and centrifugated at 300× *g* for 5 min. Following were supernatants centrifuged at 1200× *g* for 15 min, followed by 12,000× *g* for 30 min. In the end, supernatants were centrifuged at 110,000× *g* for 1 h in a Sorvall WX Ultracentrifuge Series (ThermoFisher Scientific, Waltham, MA, USA) in order to pellet exosomes. After one wash in a large volume of phosphate-buffered saline (PBS), and centrifuged at 110,000× *g* for another 1 h. At last, exosomes were resuspended in PBS (50 µL) for further analysis (27).

### Cellular Uptake of Vitamin C

Cancer cells (4×10^5^ cells/well in 24-well plates) were seeded and pre-cultured in conditioned medium with adjusted acidity-alkalinity (pH 6.5 or 7.5) with or without pantoprazole (100 μM) for 24 h. The acidity-alkalinity of the cell culture medium was controlled and regulated four times throughout the 24 h incubation. Subsequently, the culture medium was replaced by PBS of the same acidity-alkalinity (pH 6.5 or 7.5) containing 0.1 µCi (3.7 kBq) L-[14C]-ascorbic acid (PerkinElmer, Boston, MA, USA). After incubation at 37°C for 30 min with L-[14C]-ascorbic acid, the culture media was aspirated and the cells were washed three times with 1 ml of ice-cold PBS. Then 350 μl of 1 N NaOH was used to lyse the cells and the lysed cell samples were collected and counted by MicroBeta2 liquid Scintillation detector (PerkinElmer). One hundred microliters of the cell lysate were used for determination of the protein concentration by modified Lowry protein assay (Thermo Scientific). Finally, the uptake results were normalized as counts per minute (CPM) in relation to 100 μg of protein content (24).

### Cellular Uptake of 2-deoxy-2-[^18^F]fluoro-D-glucose (^18^F-FDG)

^18^F-FDG was synthesized at the Department of Nuclear Medicine of the First Affiliated Hospital of Sun Yat-sen University. Cells (PC3 and DU145 cells) plated in 6-well plates (2-5×10^5^ cells per well, 3 ml) were incubated with 1 µCi (37 kBq) ^18^F-FDG in glucose-free culture medium for 30 min at 5% CO_2_ and 37°C. After rapid washing twice with cold PBS, the cells were detached with trypsin, and cell-associated CPM was measured with a radiometric detector (PerkinElmer). Cellular uptake was expressed as the percentage of uptake per well relative to that of the control group (no treatment with vitamin C or pantoprazole).

### Animal Model: Tumor Volume and Therapeutic Regimens

Male Balb-c nude mice (4-6 weeks old, n=24) were purchased from the Model Animal Research Center of Nanjing University. For the induction of tumors, 5×10^6^ PC3 cells were suspended in sterile PBS (100 μL) and injected subcutaneously into the flank region.

Tumor diameters were measured every three days with a slide caliper. Treatments were administered when the xenografts had reached a diameter of approximately 6 mm. PC3-bearing animals were intraperitoneally injected with vitamin C (4 g/kg, twice daily), pantoprazole (200 mg/kg, daily) or a combination of vitamin C and pantoprazole. Pantoprazole was administered one day before vitamin C injection.

All mice were sacrificed 2 weeks after the initiation of treatment. After sacrifice, the tumors were dissected for immunohistochemistry (IHC). No adverse effects were observed in the animals.

### PET Imaging

^18^F-FDG (see above for synthesis) was administered *via* tail vein injection (100 μL) at an activity dose of 100 µCi (3.7 MBq) per mouse one day before and two weeks after initiation of treatment. Imaging was conducted using a micro-PET system (Inveon, SIEMENS, Germany), and the radiotracer was allowed to accumulate in the tumor for 45 min. The mice were then imaged for a 15 min static acquisition (28).

### PET Data Analysis

Tumor-to-background ratios (TBRs) were calculated to semi-quantitatively analyse^18^F-FDG uptake in the tumor. Circular three-dimensional regions of interest (ROIs) were delineated manually in the area with the highest tumor activity. The diameter did not cover the entire tumor to avoid partial volume effects. For determination of background activity, three-dimensional ROIs were delineated in the femoral muscle. The TBR was calculated using the following quotient: mean tracer uptake in the tumor/mean tracer uptake in the muscle.

### Histologic and Immunohistochemical Analysis

Tumor tissues were collected for IHC at the end of treatment. Apoptosis and proliferation were analysed based on staining with antibodies targeting Ki-67 and cleaved caspase3 (Sevicebio, Palo Alto, CA, USA) staining. Cells expressing Ki-67 or cleaved caspase3 were quantified based on H-scores. H-scores are used to assess the extent of nuclear immunoreactivity of steroid receptors. The H-score was calculated as follows:

3 * the percentage of strongly stained nuclei + 2 * the percentage of moderately stained nuclei + the percentage of weakly stained nuclei. The range of H-scores is 0 to 300. IHC analysis was performed as reported previously (10).

### Statistical Analysis

For all analyses, p < 0.05 *, < 0.01 ** or < 0.001 *** was considered as statistically significant. All analyses were performed in GraphPad Prism (GraphPad Software, Inc.). Data are presented as the mean ± SD. For each experiment, n indicates the number of individual biological repeats. For all *in vitro* and *ex vivo* studies, ≥3 technical replicates were used for each biological repeat.

## Results

### Pantoprazole Enhances the Cytotoxicity of Vitamin C and Increases Intracellular ROS Accumulation in Prostate Cancer Cells in a pH- and Time-Dependent Manner

**Figure 1:**
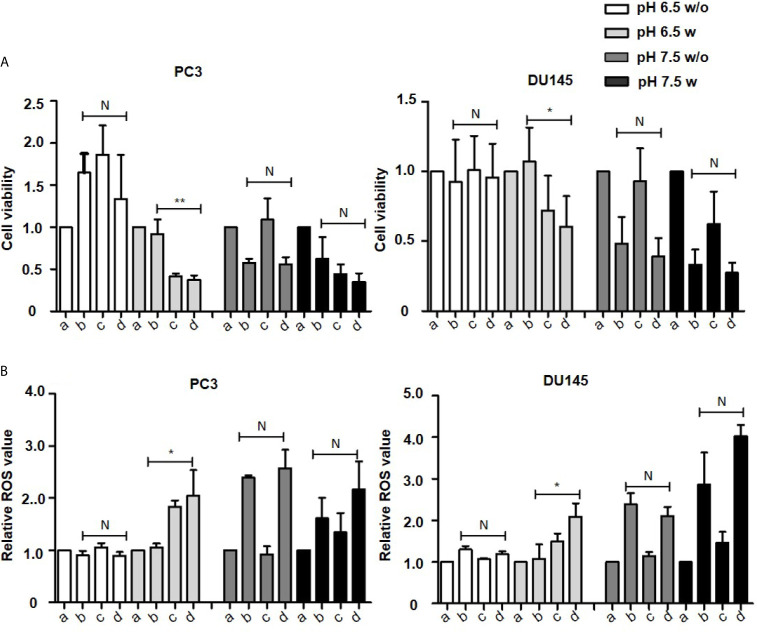
Pantoprazole in combination with vitamin C inhibits cell proliferation and induces ROS accumulation. A total of 1x10^4^ PC3 or DU145 cells per well (96-well plate) were incubated at 37°C with control (a), 4 mM vitamin C (b), 100 µM pantoprazole (c) or the combination of vitamin C and pantoprazole (d) for 4 h. Vitamin C was administered to cells with or without pretreatment with pantoprazole for 24 h (w: with pantoprazole pretreatment; w/o: without pantoprazole pretreatment). **(A)** Cell viability as assessed by the WST-8 assay and **(B)** ROS levels as detected based on fluorescence intensity 1 h to 2 h after the addition of diluted ROS detection reagent to the cell culture medium. The cell viability and ROS levels in the control group (a) were defined as 1.0. Any changes in cell viability and ROS levels following the different treatments are shown relative to the levels in the control group at two different pH values (pH 6.5 and 7.5, columns with different shades of grey). The bars represent the mean and SD of the mean of n≥3. N, not significant; *p < 0.05; **p < 0.01.

As determined by the WST-8 assay, in cell culture medium with an acidic pH (6.5), the proliferation of most cancer cells except OVCAR3 cells was unaffected by the combination of vitamin C and pantoprazole when the compounds were administered simultaneously (**Figure 1A** and **Supplement 1**). However, when cells were pretreated with pantoprazole for 24 h, pantoprazole single treatment caused a reduction in the viability of PC3 prostate cancer cells to 0.4 units and in DU145 prostate cancer cells to 0.7 units (**Figure 1A**). Moreover, the reduction in cell viability was slightly more robust following combined administration of vitamin C and pantoprazole in both prostate cancer cell lines (**Figure 1A**). In contrast, at an alkaline pH (7.5), both vitamin C single treatment and in combination with pantoprazole resulted in significant reduction of the viability in PC3 and DU145 cells. Compared to no pretreatment, pretreatment with pantoprazole (24 h) followed by combined administration of vitamin C and pantoprazole caused an additional reduction in the viability of prostate cancer cells (**Figure 1A**). Similar results were obtained for MCF7 and SKBR3 and SKOV3 cells. OVCAR3 showed somewhat different results (**Supplement 1**).

Vitamin C and pantoprazole are reported to be important for ROS production (29–31). Our results have presented that in slightly acidic cell culture medium(pH=6.5), ROS content was increased to 2.1 relative units in PC3 and to 2.2 relative units in DU145 cells following combined treatment after pretreatment with pantoprazole for 24 h. No increase was observed without pantoprazole pretreatment (**Figure 1B**). In cell culture medium with a slightly alkaline pH (7.5), the enhancement of ROS accumulation from pantoprazole was not so significant as under acidic conditions (**Figure 1B** and **Supplement 2**).

### Pantoprazole Enhances Apoptotic Cell Death, Probably Due to the Increase in Cellular Uptake of Vitamin C as Well as the Inhibition of Exosome Production by Regulation of in Intra- and Extracellular pH Values in Cancer Cells

**Figure 2:**
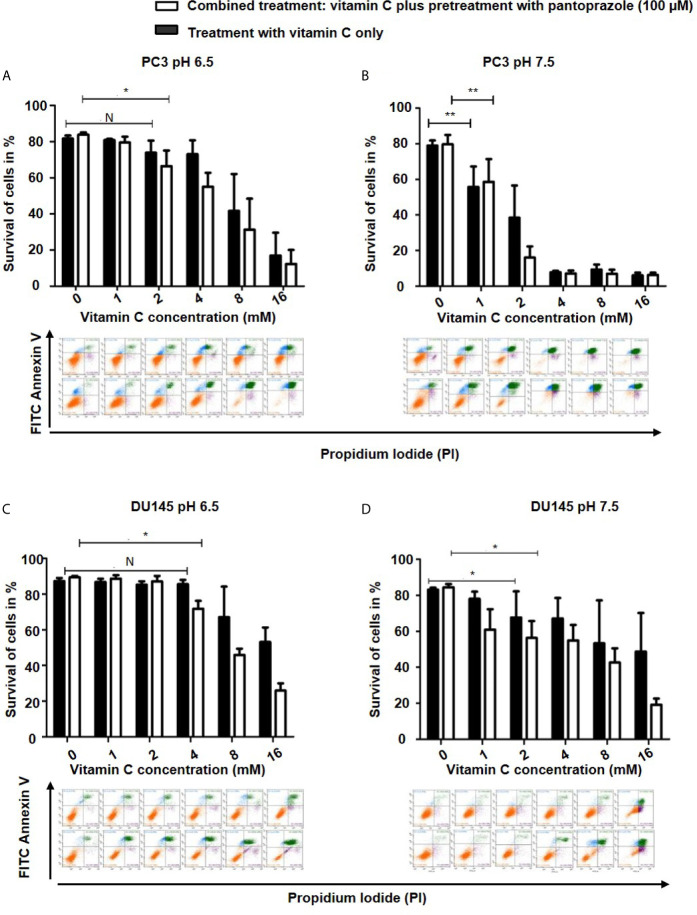
Pantoprazole in combination with vitamin C induces apoptosis of prostate cancer cells. A total of 5x10^5^ PC3 or DU145 cells per well (6-well plate) were incubated at 37°C in slightly acidic (pH 6.5) or slightly alkaline (pH 7.5) cell culture medium with different concentrations of vitamin C for 16 h following with or without pretreatment with pantoprazole (100 µM) for 24 h: Cells were incubated in cell culture medium with a pH of 6.5 (**A**: PC3 cells; **C**: DU145 cells) or a pH of 7.5 (**B**: PC3 cells; **D**: DU145 cells). Column diagram (upper panel): quantification of the FACS results. Colorized dot plot (bottom panel): FACS analysis data with increasing vitamin C concentrations as shown in the column diagrams (orange: surviving cells; green: PI- and AV- positive cells [apoptotic cells]; blue: PI-positive cells [necrotic cells]). Upper rows of the colorized dot plots: cells were treated with vitamin C only; bottom rows of the colorized dot plots: cells were treated with vitamin C and pantoprazole. The bars represent the mean and SD of the mean of n≥3. N, not significant; *p < 0.05, **p < 0.01.

To characterize the cytotoxic mechanism of vitamin C and pantoprazole in cancer cells, we first monitored apoptotic cell death using flow cytometric analysis (FACS). FACS analysis revealed that pantoprazole enhanced vitamin C-induced apoptotic cell death, as shown by a significant increase in the number ofannexin V/PI-positive cells as well as a marked decrease in the number of live, annexin V/PI-negative cells. This was observed in PC3 and DU145 cells at a slightly acidic pH (6.5) (**Figures 2A, C**). For example, after 16 h of treatment with 4 mM vitamin C, 59% of PC3 cells and 58% of DU145 cells survived following treatment with vitamin C plus pretreatment with pantoprazole (100 µM), whereas 74% of PC3 cells and 70% of DU145 cells survived following treatment with vitamin C only (**Figures 2A, C**). In cell culture medium with a pH of 7.5 (slightly alkaline), a similar effect was found in DU145 cells following combined treatment. However, in PC3 cells, particularly at vitamin C concentrations of 4, 8 and 16 mM, the elimination of tumors cells induced by the combined treatment regimen (vitamin C plus pretreatment with pantoprazole) was not superior to that with vitamin C only (**Figures 2B, D**). FACS analysis of breast and ovarian cancer cells also showed that the synergistic effect of pantoprazole on cytotoxicity in slightly acidic (pH 6.5) cell culture medium was stronger than that in alkaline (pH 7.5) cell culture medium (**Supplement 3**).

**Figure 3:**
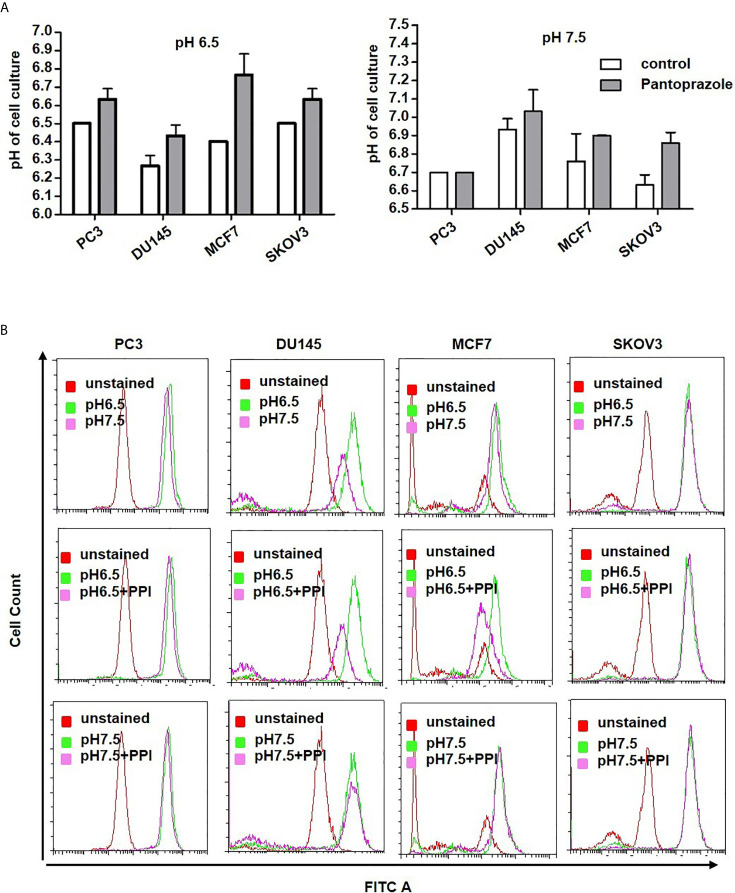
Pantoprazole regulates the extra- and intracellular pH of cancer cells. **(A)** Change in the extracellular pH of the cell culture medium of prostate (PC3, DU145), breast (MCF7) and ovarian cancer (SKOV3) cells following treatment with pantoprazole (grey columns) or without pantoprazole (controls, white columns) at a pH of 6.5 or 7.5. **(B)** Change in the intracellular pH of prostate (PC3 and DU145), breast (MCF7) and ovarian cancer (SKOV3) cells following treatment with or without 100 μM pantoprazole for 6 h at a pH of 6.5 or 7.5. Top panel: cells were incubated in cell culture medium of different pHs; middle panel: cells were incubated in cell culture medium with a pH of 6.5 with or without pantoprazole; bottom panel: cells were incubated in cell culture medium with a pH 7.5 with or without pantoprazole. Red pick: unstained cells; pink pick: cells incubated in cell culture medium with a pH of 7.5 containing pantoprazole; green pick: cells incubated in cell culture medium with a pH of 7.5 without pantoprazole; X-axis: FITC; Y-axis: cell count. The bars represent the mean and SD of the mean of n≥3.

**Figure 4:**
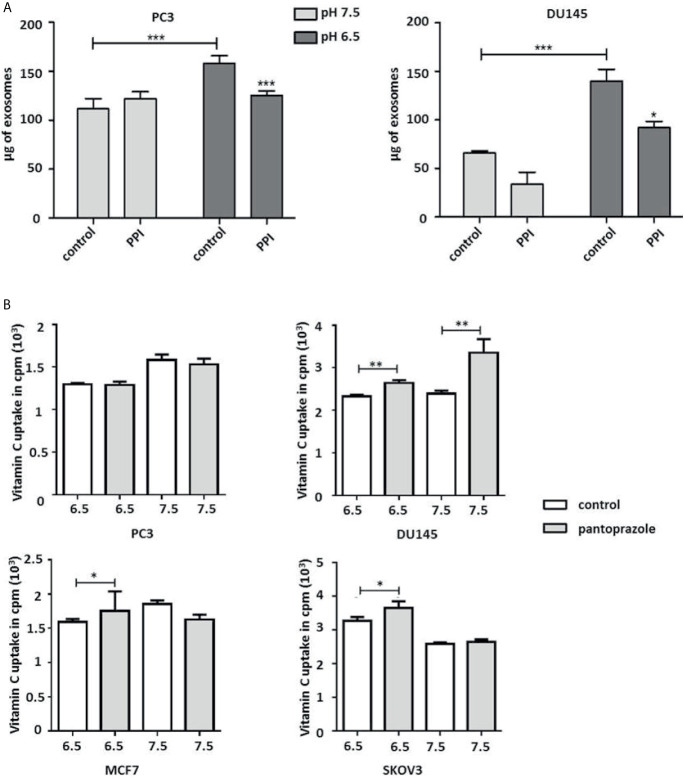
Pantoprazole significantly increases cellular vitamin C uptake and inhibits the production of exosomes depending on the pH value of the cell culture medium. **(A)** PC3 and DU145 cells were cultured in cell culture medium with a pH of 7.5 or a pH of 6.5 and treated with pantoprazole (100 μM) (PPI) or left untreated (control). The protein concentration was determined by the BCA protein assay, and exosomes were lysed using RIPA buffer. **(B)** Vitamin C uptake in prostate (PC3 and DU145), breast (MCF7) and ovarian cancer cells (SKOV3) following treatment with 100 μM pantoprazole for 6 h (grey columns) or without pantoprazole (control, white columns) in cell culture medium with a pH of 6.5 or 7.5. The bars represent the mean and SD of the mean of n≥3. *p < 0.05; **p < 0.01, ***p < 0.001.

Our results also demonstrated that pantoprazole slightly increased the extracellular pH of the cell culture medium at both pH 6.5 and pH 7.5 (**Figure 3A**). Moreover, the intracellular pH of prostate and breast cancer cells was modified following alteration of the extracellular pH or following pantoprazole treatment (**Figure 3B**). This effect of pantoprazole seemed to be stronger in acidic (pH 6.5) cell culture medium than in alkaline (pH 7.5) cell culture medium (**Figure 3B**). However, in SKOV3 cells, we did not observed a clear change in the intracellular pH in response to pantoprazole treatment (**Figure 3B**). Furthermore, we noticed that in comparison with acidic pH (6.5), the alkaline pH (7.5) inhibited the production of exosomes significantly in both prostate cancer cell lines (**Figure 4A**). Moreover, pantoprazole reduced the secretion of exosomes under acidic (6.5) but not alkaline conditions (**Figure 4A**). We also analyzed the cellular uptake of L-[^14^C]-ascorbic acid (vitamin C) after the addition of pantoprazole (100 µM for 6 h without the administration of vitamin C) to the culture medium at a slightly acidic pH (6.5) and a slightly alkaline pH (7.5) (**Figure 4B**). In DU145 cells incubated at pH 6.5 and 7.5 and in ovarian cancer cells incubated at pH 6.5, pantoprazole induced a significant increase in vitamin C uptake. However, in PC3 no difference in cellular vitamin C uptake was observed following addition of pantoprazole at pH 6.5 or pH 7.5. The same was true for MCF7 and SKOV3 at pH 7.5 (**Figure 4B**).

We have also assayed the synergistic effect of redox-active iron with vitamin C treatment in prostate, breast and ovarian cancer cells by performing the WTS-8 assay following combined treatment with vitamin C and chelators that inhibit the redox cycling of iron (DFO and DTPA) (22, 23). Interestingly, we found that compared with control treatment, both intracellular and extracellular administration of chelators (DFO/DTPA) inhibited vitamin C-induced cytotoxicity in human prostate, breast and ovarian cancer cells (**Supplement 4**). Pantoprazole did not significantly influence the effect of chelators on the toxicity of vitamin C, although pantoprazole could promote the cytotoxicity of vitamin C (**Supplement 4**).

### Combined Treatment With Vitamin C and Pantoprazole Significantly Inhibits Tumor Growth of Prostate Cancer Xenografts

**Figure 5:**
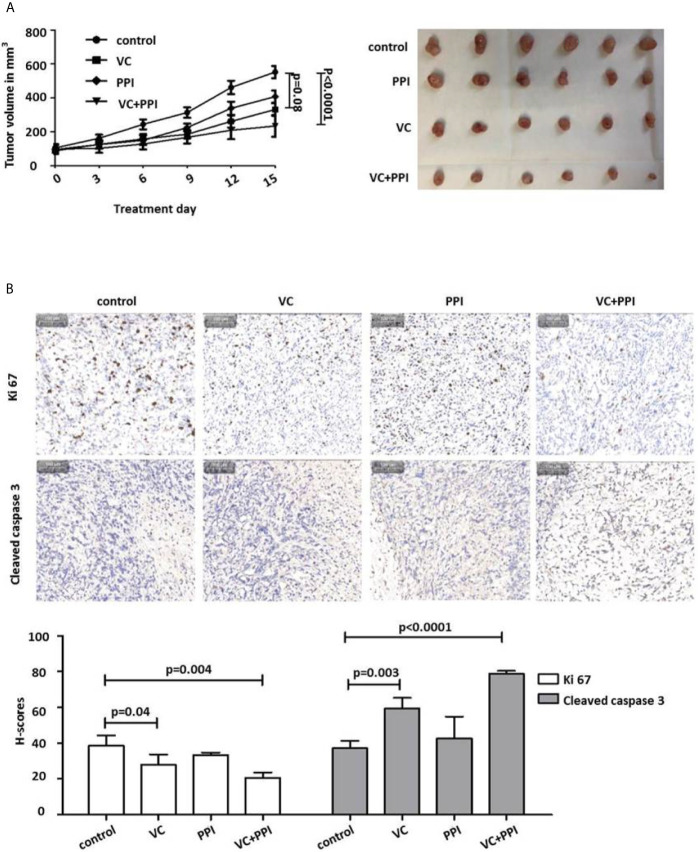
Pantoprazole enhances the anticancer effect of vitamin C in mice bearing subcutaneous PC3 xenografts. **(A)** BALB/c nude mice (n=24; 4-6-week-old males) were subcutaneously injected with 5×10^6^ PC3 cells. When the tumor size had reached 100 mm^3^, pantoprazole (PPI, 200 mg/kg, daily) and vitamin C (VC, 4 g/kg, twice daily) were injected intraperitoneally into the mice. After 15 days, all mice were euthanized, the remaining tumors were removed, and their volumes were measured. In the combined treatment group (VC + PPI), pantoprazole was given one day before injection of vitamin C. Left: tumor growth curves (day 0 – day 15) after the respective treatments; right: tumors prepared from the six different mice from each treatment group (control, PPI, VC, VC + PPI) after sacrifice (day 15). **(B)** Immunohistochemical (IHC) analysis of the proliferation marker Ki67 and the apoptosis marker cleaved caspase-3 in explanted tumors (upper panel). The grey bar in the upper left corner of each picture represents 100 µm. The lower panel shows semiquantitative analysis of the IHC results using H-scores as described in the Methods section. Quantification of the IHC results demonstrated a significant decrease (p = 0.004) in expression of the proliferation marker Ki67 and a significant increase (p < 0.0001) in expression of the apoptosis marker cleaved caspase 3 in VC + PPI-treated cells compared to controls, respectively. The bars represent the mean and SD of the mean of n≥3.

In control animals that received placebo following subcutaneous inoculation of PC3 prostate cancer cells (5×10^6^), the doubling time of the PC3 xenografts was 7 days (**Figure 5A**). Treatment was initiated in animals from the different treatment groups (VC, PPI, VC + PPI) as soon as tumors had reached a volume of approximately 100 mm^3^(**Figure 5A**). While the control group received placebo only, the VC group was injected intraperitoneally with 4 g/kg vitamin C twice daily, the PPI group received intraperitoneal injection of 200 mg/kg pantoprazole daily, and the combined group (VC+PPI) was administered 200 mg/kg pantoprazole (daily) combined with 4 g/kg vitamin C (twice daily). In the combined group, pantoprazole was administered one day before vitamin C. Combined treatment (VC+PPI) significantly suppressed tumor growth compared to the untreated control group, as observed for PC3 xenografts 15 days after initiation of therapy (p<0.0001) (**Figure 5A**). While treatment with vitamin C alone caused a slight reduction of tumor load compared to control group (p = 0.08; **Figure 5A**). Accordingly, the therapeutic efficacy of the combined regimen (VC + PPI) was significantly better than that of the control group (p<0.0001) (**Figure 5A**). In addition, treatment of tumors with the combination therapy led to more cleaved caspase-3-positive (apoptotic) cells (p < 0.0001) than treatment with vitamin C only (p = 0.003) or control treatment (**Figure 5B**). Furthermore, exposure to the combined treatment regimen significantly decreased the percentage of Ki67-positive cells from 38.5 to 20.5 (p = 0.004) (**Figure 5B**). Treatment with vitamin C only induced a comparatively lower decrease in the percentage of Ki67-positive cells (p = 0.04) (**Figure 5B**).

### Functional *In Vivo* Imaging of the Response to Combined Treatment With Vitamin C and Pantoprazole Indicates a Significant Reduction in^18^F-FDGuptake in Prostate Cancer Xenografts

**Figure 6:**
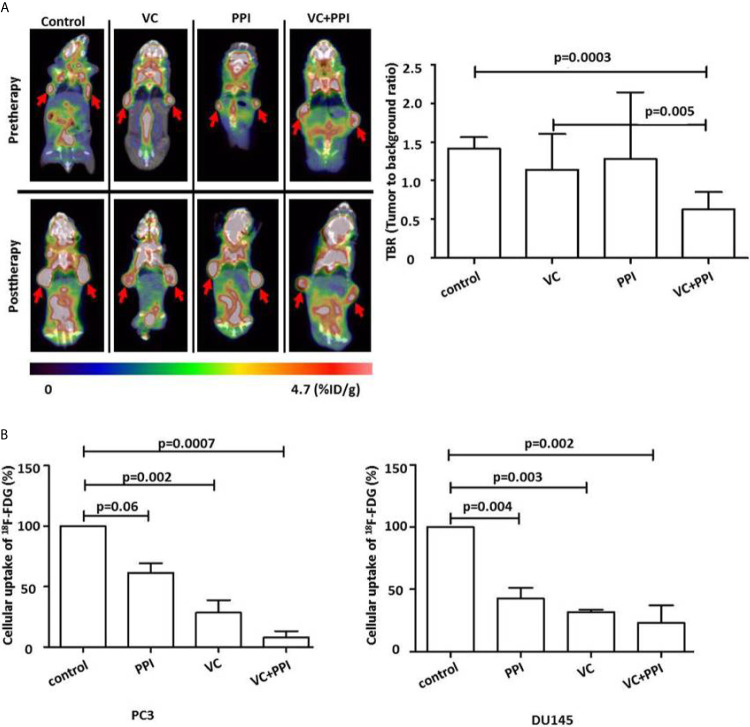
Functional FDG-PET/CT imaging showing that pantoprazole enhances the anticancer effect of vitamin C *in vivo*. **(A)** Left panel: representative PET/CT scans showing the changes in tumor uptake of ^18^F-FDG (red arrows). ^18^F-FDG-PET/CT scans were carried out before (Pretherapy, day 0) and 14 days after the initiation of treatment (Posttherapy, day 14). The red arrows indicate the sites of subcutaneous injection of PC3 tumor cells and the accumulation of ^18^F-FDG. Right panel: uptake of ^18^F-FDG in tumor tissue (tumor to background ratio [TBR]) in control mice and mice treated with vitamin C (VC), pantoprazole (PPI) or both (VC+PPI). The TBR was calculated 14 days after the initiation of treatment and served as an indicator of tracer uptake. The mean TBR as detected via^18^F-FDG-PET/CT was significantly reduced following combined treatment (VC + PPI) compared with control treatment (p = 0.0003), indicating a significant reduction in tumor volume. Moreover, the reduction in TBR after combined VC + PPI treatment compared to treatment with vitamin C only was also significant (p = 0.005). **(B)** PC3 and DU145 prostate cancer cells were incubated with ^18^F-FDG for 30 min at 5% CO_2_ and 37°C. After washing twice with cold PBS, the cells were detached with trypsin, and cell-associated ^18^Fradioactivity was quantified with a γ-counter. ^18^Fradioactivity is expressed as percent uptake per cell relative to the uptake of untreated controls. In both prostate cancer cell lines (PC3 and DU145), the combination treatment significantly reduced cellular uptake of ^18^F-FDG (p=0.0007 in PC3 and p=0.002 in DU145, compared to control, respectively. VC: treatment with vitamin C (4 mM); PPI: treatment with pantoprazole (100 µM); VC + PPI: combined treatment with vitamin C (4 mM) and pantoprazole (100 µM). The bars represent the mean and SD of the mean of n≥3.

^18^F-FDG-PET imaging was used to evaluate early responses. Mice bearing PC3 xenografts were treated as descript before. For ^18^F-FDG-PET monitoring, the mice were injected intravenously with 3.7 MBq (100 µCi) ^18^F-FDG.As depicted in **Figure 6A**, tumor growth was significantly inhibited by the combined treatment. That is, ^18^F-FDG-PET imaging showed a significant reduction in^18^F-FDG uptake at 14 days after treatment initiation (post therapy) compared with pretreatment values (pre therapy) (**Figure 6A**). Additionally, combined treatment with VC + PPI *in vitro* induced a stronger reduction in cellular ^18^F-FDGuptake (PC3: p=0.0007, DU145:p=0.002) in both prostate cancer cell lines than treatment with vitamin C alone (PC3: p=0.06, DU145: p=0.004) or pantoprazole alone (PC3: p=0.002, DU145:p=0.003), indicating a reduced growth of tumor cells (**Figure 6B**).

## Discussion

Previous studies have demonstrated that vitamin C triggers cancer cell-selective cytotoxicity *in vitro* (18, 32, 33). Our previous study has shown the impact of pH on the anticancer effect of vitamin C in PC3 and DU145 prostate cancer cells (24). Moreover, we could show that treatment of prostate cancer cells with vitamin C induces pH-dependent apoptosis through the generation of ROS as well as a reduction in NADPH levels *in vitro* (24). Additionally, Ngo et al. discussed how vitamin C is beneficial to anticancer therapy by targeting three vulnerabilities of cancer cells such as: redox imbalance, epigenetic reprogramming and oxygen-sensing regulation (34). They also summarized clinical trials with regarding to this anticancer feature of vitamin C in order to develop more effective combination strategies and improve the overall clinical study design in the future (34).

Proton pump inhibitors (PPIs) have shown to be beneficial for cancer chemoprevention by reduction of proliferation and induction of apoptosis in multiple cancer cell lines (11, 12, 35). PPIs are commonly used to treat acid-related diseases, might promote the disruption of pH homeostasis in tumor cells by targeting V-ATPase (11, 16, 26). PPIs have also been reported to enhance a pH dependent anticancer effect on cancer cells through regulation of the production of exosomes and the extracellular pH, which regulates the production of exosomes involved in the pathogenesis of cancers (25, 27, 36). The identical change of exosome production was found from our experiments (**Figure 4A**). In the current study, we have demonstrated that the PPI pantoprazole increases the cytotoxicity of vitamin C in the treatment of metastatic castration-resistant prostate cancer *in vitro* and *in vivo*. Our results also highlight the regulation of pH in the tumor microenvironment (**Figure 3**), ROS accumulation (**Figure 1** and **Supplements 1**, **2**) and exosome production (**Figure 4A**) following combined anticancer treatment with vitamin C and pantoprazole *in vitro*.

Furthermore, a series of clinical studies on the administration of PPIs to patients suffering from different cancers demonstrated that PPIs may be new effective agents for anticancer therapy (37–39). In addition, most patients suffering from CRPC undergo a long-term therapeutic regimen; thus low-income individuals may have unable to receive further adequate anticancer treatment. Drug repurposing supplies a cheaper and probably more efficient therapeutic possibility (9). Previous studies showed that repurposing PPIs could enhance the efficacy and safety of chemotherapy as well as improve the therapy in solid tumors (40, 41). We have observed a stronger therapeutic effect when cancer cells were pretreated with pantoprazole for 24 h than after simultaneous treatment vitamin C and pantoprazole simultaneously (**Figure 1**, **2** and **Supplements 1**, **2**). This could be explained with the fact that pantoprazole pretreatment significantly increased vitamin C uptake in DU145, MCF7 and SKOV3 cells (**Figure 4B**). However, pantoprazole pretreatment did not significantly increase the uptake of vitamin C in cells incubated in cell culture medium with a pH of 7.5. This might due to that the therapeutic effect of PPIs is pH dependent (27). Our results have also shown that pantoprazole had a more beneficial anticancer effect in a slightly acidic environment (**Figure 1** and **Supplements 1**, **2**). Pantoprazole significantly enhanced the cellular uptake of vitamin C in cells incubated in slightly acidic cell culture medium (pH 6.5). Furthermore, the toxicity of vitamin C in NSCLC and GBM has been reported to depend on redox-active labile iron (22). Likewise, we have demonstrated that in prostate cancer cells (PC3, DU145), breast cancer cells (MCF7) and ovarian cancer cells (SKOV3), the cytotoxicity of vitamin C depends on redox-active labile iron (**Supplement 4**). Nevertheless, pantoprazole induces the enhancement of cellular toxicity of vitamin C. However, pantoprazole has no additional influence on iron redox cycling in cancer cell lines (**Supplement 4**).

Recent studies have demonstrated that PET/CT imaging using PSMA ligands provides a higher sensitivity and specificity than other imaging methods for the evaluation of advanced prostate cancer (6, 7, 42). However, in China, PET/CT imaging using PSMA ligands is still not an established approach for the preclinical and clinical detection of prostate cancer. Other Studies have suggested that ^18^F-FDG PET might be useful for monitoring and predicting the therapeutic response to androgen deprivation therapy in patients with metastatic prostate cancer (43, 44). PC3 cells isolated from metastatic prostate cancer patients have been reported to be PSMA-negative and castration-resistant (45, 46). In this study, we transplanted PC3 cells into mice to mimic mCRPC, treated the mice with vitamin C and/or pantoprazole and monitored the therapeutic effect of these drugs using ^18^F-FDG PET/CT imaging. We could identify the location of the mCRPC (PC3) xenografts. Furthermore, we have demonstrated that ^18^F-FDGPET/CT imaging allows monitoring of the therapeutic response following combined treatment with vitamin C and pantoprazole. As we could show, treatment induced a significant reduction in^18^F-FDG uptake in prostate cancer xenografts after two weeks (**Figure 6A**). Therefore, our data support the further clinical investigation of ^18^F-FDG PET/CT imaging for the prediction of therapeutic responses in patients with castration-resistant metastatic prostate cancer following combined therapy with pantoprazole and vitamin C.

## Conclusion

The drug repurposing of pantoprazole and vitamin C seems to be an alternative therapeutic strategy for patients suffering from mCRPC, since therapy using PSMA I&T or PSMA-617 labelled with Lu-177 or Ac-225 is not available worldwide. We have shown that pantoprazole enhances the anticancer effect of vitamin C in prostate cancer cells by increasing cellular vitamin C uptake, inhibiting exosome production and altering the intracellular and extracellular pH. Moreover, ^18^F-FDG-PET proved to be useful for monitoring the therapeutic response in CRPC.

## Data Availability Statement

The original contributions presented in the study are included in the article/**Supplementary Material**. Further inquiries can be directed to the corresponding authors.

## Ethics Statement 

The animal study was reviewed and approved by The Institutional Animal Care and Use Committee of The First Affiliated Hospital of Sun Yat-sen University.

## Author Contributions

Conceptualization, ZL and XZ. Data curation, ZL, PH, YL, CS, and XZ. Formal analysis, ZL, PH, YL, WS, ZC, BZ, CS and XZ. Funding acquisition, ZL, DY, and XZ. Investigation, ZL, PH, YL, GY, ZC, BZ, YW, and XZ. Methodology, ZL, PH, YL, GY, WS, ZC, BZ, and XZ. Project administration, ZL and XZ. Resources, ZL, DY, CS, and XZ. Supervision, ZL and XZ. All authors contributed to the article and approved the submitted version.

## Funding

This research was funded by the National Science Foundation for Young Scientists of China [grant number 81901793], the Young Teacher Foundation of Sun Yat-sen University (CN) [grant number 19ykpy55], the Science and Technology Program of Guangzhou [grant number 201607010353] and the Science and Technology Program of Guangzhou [grant number 201707010110]. These funding programs finically supported our study.

## Conflict of Interest

The authors declare that the research was conducted in the absence of any commercial or financial relationships that could be construed as a potential conflict of interest.

## Abbreviations

BSA, bovine serum albumin; CRPC, castration-resistant prostate cancer; DBA, dolichosbifows agglutinin; DFO, desferrioxamine; DHA, dehydroascorbate; DTPA, diethylenetriamine-pentaacetic acid; ECM, extracellular matrix; FACS, fluorescence-activated cell sorting; FDG, fluorodeoxyglucose; GLUTs, glucose transporters; GBM, glioblastoma multiforme; LNCaP, androgen-sensitive human prostate adenocarcinoma; LIP, labile iron pool; MDR, metastasis and multidrug resistance; mCRPC, metastatic castration-resistant prostate cancer; NSCLC, non-small cell lung cancer; NSAID, non-steroidal anti-inflammatory drug; PBS, phosphate buffered solution; PET, positron emission tomography; PI, propidium iodide; PPI, proton pump inhibitor; RCTs, randomized controlled clinical trials; ROIs, regions of interest; ROS, reactive oxygen species; SVCTs, sodium-dependent vitamin C transporters; TBR, tumor to background ratio; VC, vitamin C.
