# Supplementary material for: Drug Repurposing of Pantoprazole and Vitamin C Targeting Tumor Microenvironment Conditions Improves Anticancer Effect in Metastatic Castration-Resistant Prostate Cancer
Source: Front Oncol. 2021 Jul 7;11:660320. doi: 10.3389/fonc.2021.660320 (PMC8294332; doi:10.3389/fonc.2021.660320)
Supplement: Supplementary file 1 [file DataSheet_1.doc]

Supplementary Material

## Supplementary Figures

#
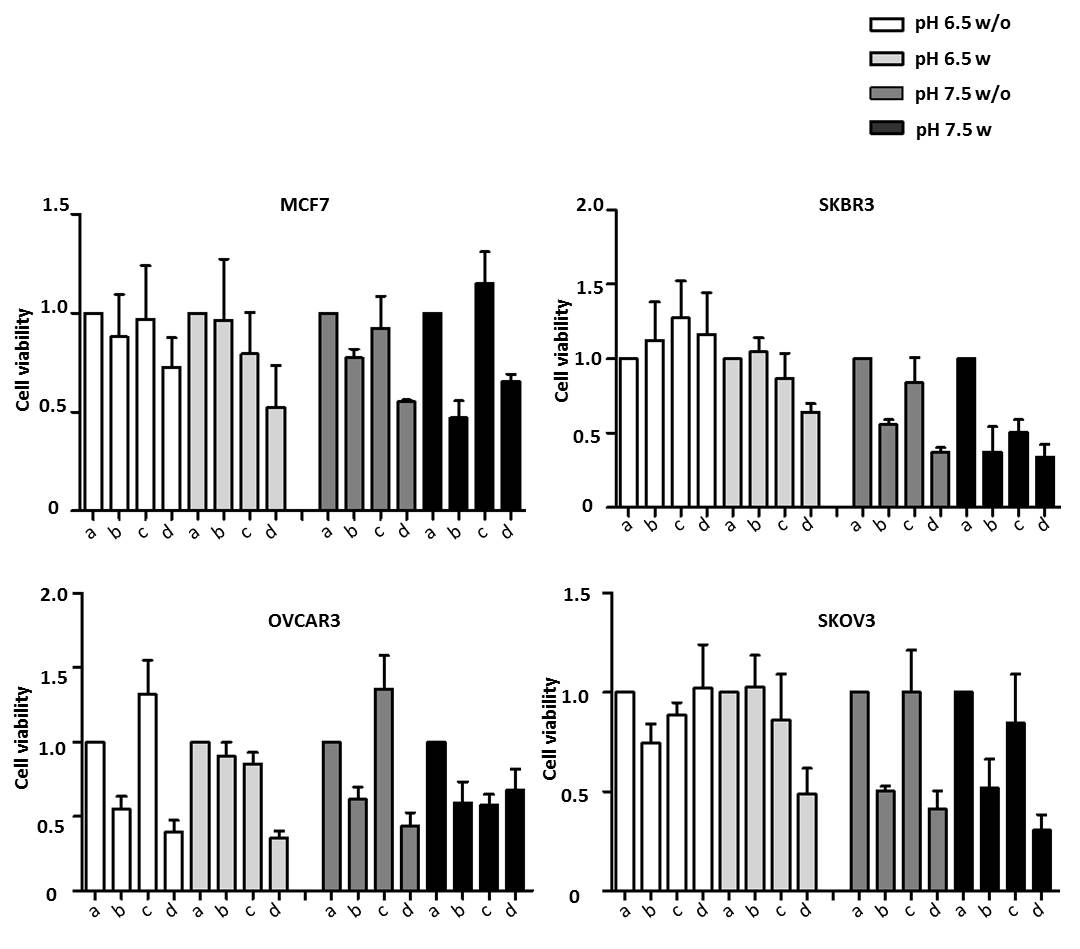


# Supplement 1. Pantoprazole in combination with vitamin C inhibits cell proliferation.A total of 1x10^4^ cancer cells per well (96-well plate) were incubated at 37°C with 4mM vitamin C, 100 µM pantoprazole or both for 4 h. Vitamin C was administered to cells with or without pretreatment with pantoprazole for 24 h (w: with pretreatment of pantoprazole for 24 h; w/o: without pretreatment of pantoprazole for 24 h, at both pH 6.5 and pH 7.5). Cell viability was assessed by the WST-8 assay (a:control; b: treated with vitamin C; c: treated with pantoprazole; d: treated with the combination of 4 mM vitamin C and 100 µM pantoprazole). The bars represent the mean and SD of the mean of n≥3.

#
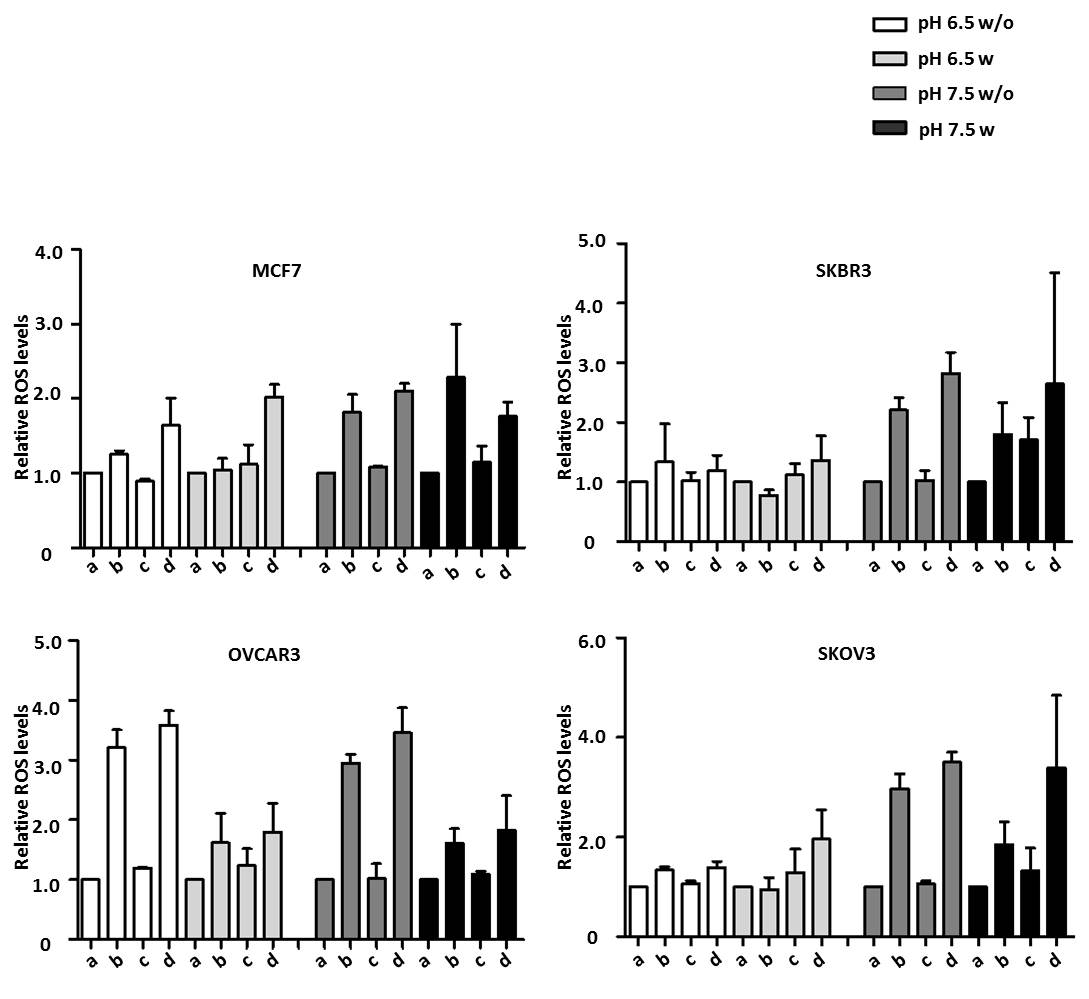


# Supplement 2.Pantoprazole in combination with vitamin C induces ROS accumulation in cancer cells. A total of 1x10^4^ cancer cells per well (96-well plate) were incubated at 37°C with 4 mM vitamin C, 100 µM pantoprazole or both for 4 h. Vitamin C was administered to cells with or without pretreatment with pantoprazole for 24 h (w: with pretreatment of pantoprazole for 24 h; w/o: without pretreatment of pantoprazole for 24 h, at both pH 6.5 and pH 7.5). ROS levels were then detected 1 h after the addition of ROS detection reagent to the cell culture medium. The ROS level in the control group was set as 1, and changes in ROS levels compared with the level in the control group are shown for cells incubated under different conditions (a:control; b: treated with 4mM vitamin C; c: treated with 100 µM pantoprazole; d: treated with the combination of 4 mM vitamin C + 100 µM pantoprazole). The bars represent the mean and SD of the mean of n≥3.

#
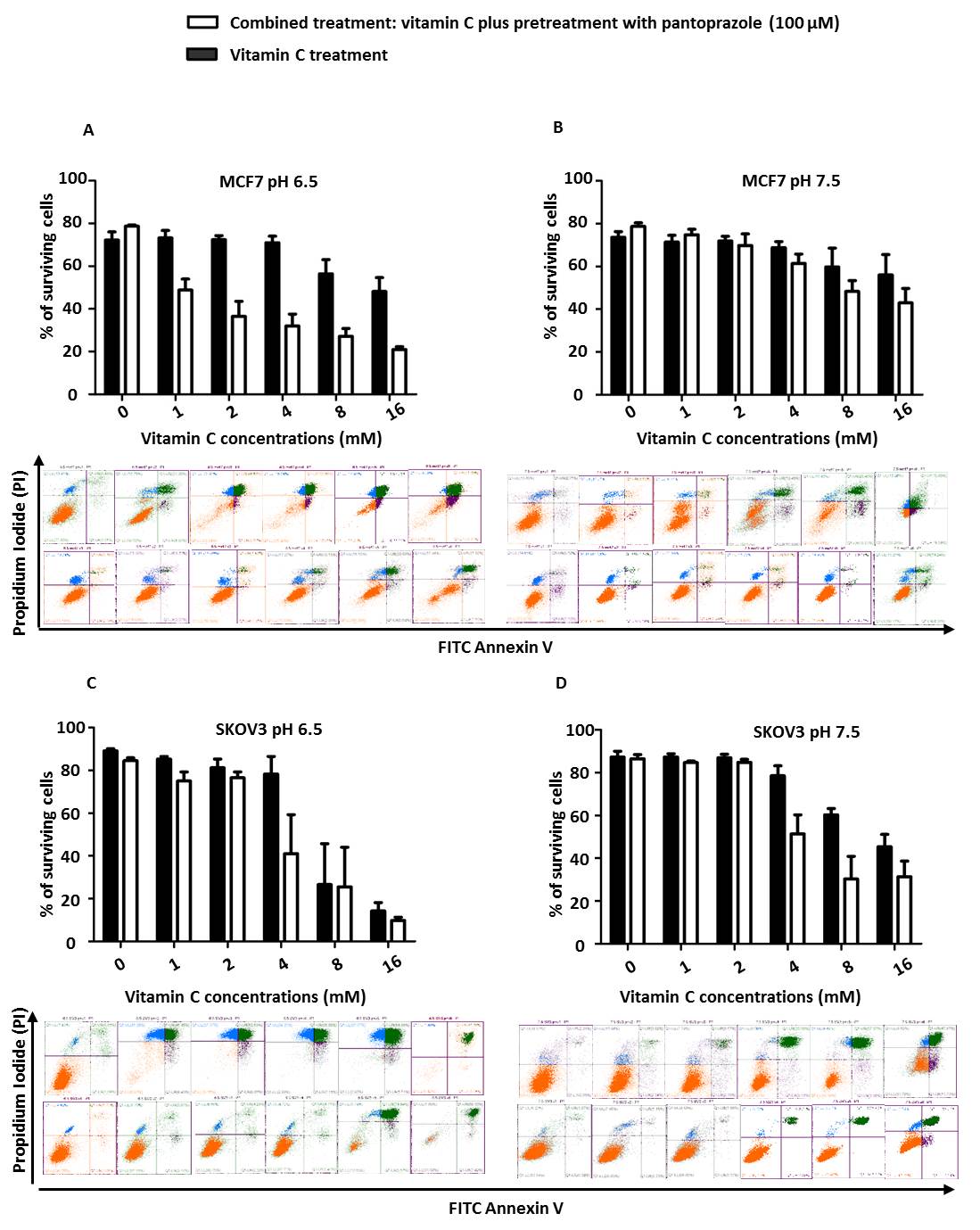


# Supplement 3. Pantoprazole in combination with vitamin C induces apoptosis of cancer cells. A total of 4x10^5^ MCF7 or SKOV3 cells per well (6-well plate) were incubated at 37°C in slightly acidic (pH 6.5) or slightly alkaline (pH 7.5) cell culture medium with different concentrations of vitamin C for 16 h and pretreated with or without pantoprazole (100 µM) for 24 h (MCF7 cells were incubated in cell culture medium with a pH of 6.5 (A) or a pH of 7.5 (B), and SKOV3 cells were incubated in cell culture medium with a pH of 6.5 (C) or a pH of 7.5 (D)). The bars represent the mean and SD of the mean of n≥3.

# Upper panels (column graphs) show the survival of cells, as determined by the FACS data shown in the lower panels (colourized dot plots; orange: surviving/alive cells (FITC-Annexin V-negative and PI-negative), green: PI- and AV-positive cells (apoptotic cells); blue: PI-positive cells (necrotic cells)). Upper rows of the colourized dot plots: cells treated with 4mM vitamin C +100 µM pantoprazole bottom rows of the colourized dot plots: cells treated with 4mM vitamin C only. The bars represent the mean and SD of the mean of n≥3.

#
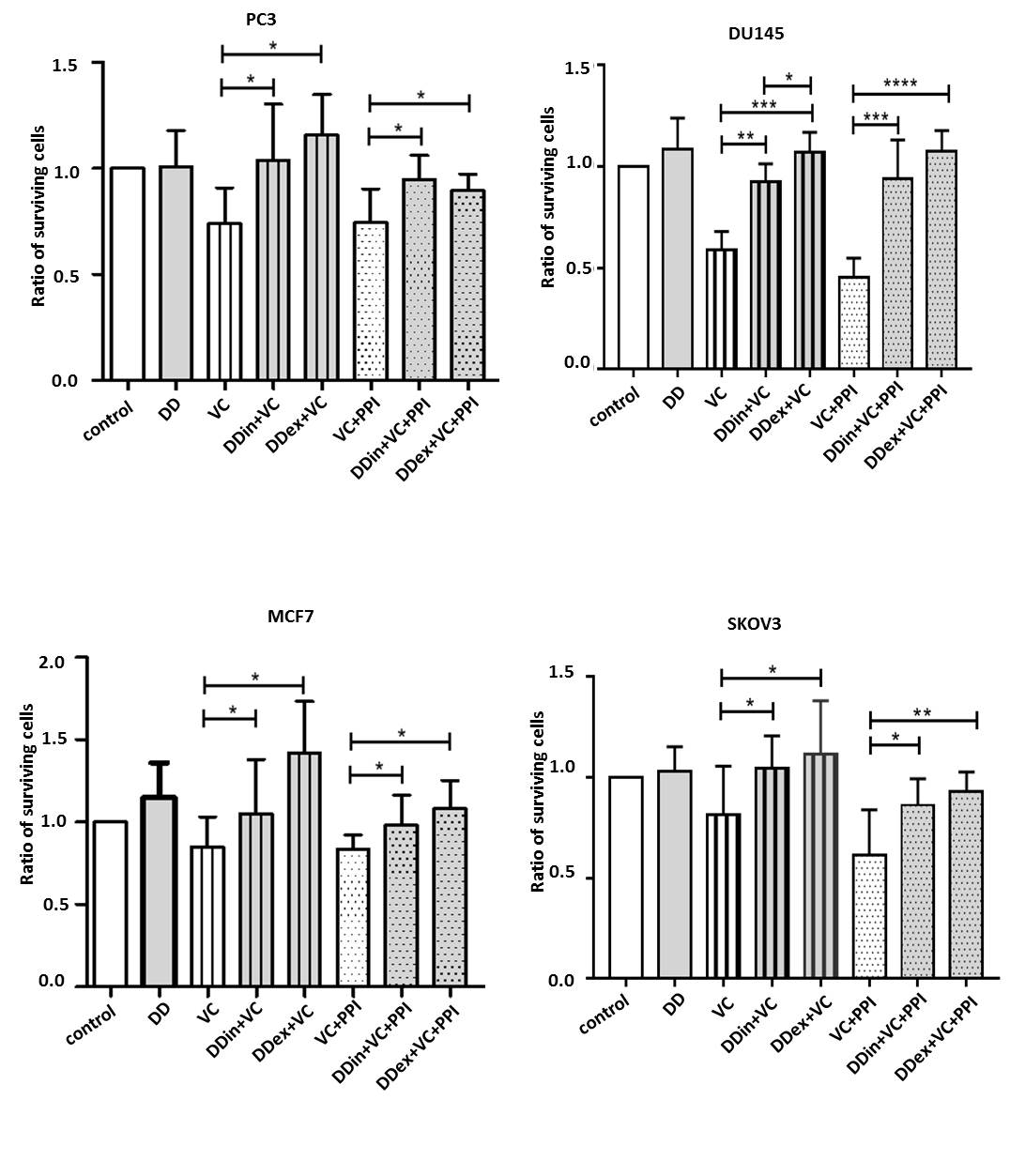


# Supplement 4. Iron redox cycling effectors can improve the effect of vitamin C treatment *in vitro*.

# Prostate (PC3 and DU145), breast (MCF7) and ovarian (SKOV3) cancer cells were treated as follows:

# Control: without treatment;

# DD: treatment with 200 μM DFO + 1 mM DTPA for 3 h;

# VC:treatment with vitamin C (4 mM) for 1;

# VC + PPI: treatment with vitamin C (4 mM) for 1; pretreatment with pantoprazole (100 μM) for 24 h.DDin + VC + PPI: intracellular treatment with 200 μM DFO + 1 mM DTPA for 3 h followed by washing with cell culture medium (37°C) prior to vitamin C exposure (4 mM, 1 h); pretreatment with pantoprazole (100 μM) for 24 h.

# DDex + VC + PPI: the cell culture medium was prepared for 3 h at 37°C in the absence of cells, and then the cells were treated with 200 μM DFO + 1 mM DTPA and exposed to vitamin C (4 mM) for 1 h (ex = extracellular); pretreatment with pantoprazole (100 μM) for 24 h.

# The bars represent the mean and SD of the mean of n=3. *: p<0.05; **: p<0.01; ***: p<0.001.
